# Levodopa/Benserazide Loaded Microspheres Alleviate L-dopa Induced Dyskinesia through Preventing the Over-Expression of D1R/Shp-2/ERK1/2 Signaling Pathway in a Rat Model of Parkinson's Disease

**DOI:** 10.3389/fnagi.2017.00331

**Published:** 2017-10-18

**Authors:** Ying Wan, Na Wu, Lu Song, Xijin Wang, Zhenguo Liu, Weien Yuan, Jing Gan

**Affiliations:** ^1^Department of Neurology, Xinhua Hospital Affiliated to Shanghai JiaoTong University, School of Medicine, Shanghai, China; ^2^School of Pharmacy, Shanghai Jiao Tong University, Shanghai, China

**Keywords:** L-dopa induced dyskinesia, D1R, Shp-2, 6-OHDA, continuous dopamine stimulation, microspheres

## Abstract

**Background:** The long-term intermittent Levodopa (L-dopa) stimulation contributes to an aberrant activation of D1 receptor (D1R) mediated extracellular signal-regulated kinases1/2 (ERK1/2) in the striatal medium spiny neurons, resulting in the occurrence of L-dopa induced dyskinesia (LID). Recently, a novel signaling pathway, D1R/Shp-2/ERK1/2, was proposed to be required for the occurrence of LID. Here we designed the study in which two different methods of L-dopa delivery [continuous dopamine stimulation (CDS) vs. intermittent dopamine stimulation] were used to further identify: (1) the role of D1R/Shp-2/ERK1/2 signaling pathway in the occurrence of LID; (2) whether CDS alleviated LID though preventing the over-expression of the D1R/Shp-2/ERK1/2 signaling pathway.

**Methods:** 6-OHDA-lesioned rat models of Parkinson's disease (PD) were randomly divided into two groups to receive intermittent L-dopa stimulation (L-dopa/benserazide standard group, LS group) or CDS (L-dopa/benserazide loaded microspheres, LBM group) for 21 days. Dyskinesia and anti-parkinsonian effect were compared between the two groups through the AIMs assessment and cylinder test. The critical protein changes in the D1R/Shp-2/ERK1/2 signaling pathway were compared between the two groups through Western blotting.

**Results:** Intermittent L-dopa administration induced serious dyskinetic movements in the 6-OHDA-lesioned rats, and the anti-parkinsonian effect of L-dopa was gradually counteracted by the occurrence of dyskinesia. Intermittent L-dopa administration enhanced the expression of membrane D1R, and induced a robust increase of phosphorylation of Shp-2, Src, DARPP-32, and ERK1/2 in the 6-OHDA-lesioned striatum. In contrast, CDS played a dose-dependent anti-parkinsonian role, without inducing such apparent dyskinetic movements. Moreover, CDS induced no change of membrane D1R expression or phosphorylation of Shp-2, Src, DARPP-32, and ERK1/2 in the 6-OHDA-lesioned striatum.

**Conclusion:** The aberrant activation of D1R/Shp-2 complex was evidenced to be required for the D1R mediating ERK1/2 phosphorylation and the occurrence of LID. CDS effectively prevented the overexpression of D1R/Shp-2/ERK1/2 signaling pathway, resulting in the reduction of LID in 6-OHDA-lesioned rats model of PD.

## Introduction

Parkinson's disease (PD) is one of the most common neurodegenerative diseases in aged people. The disease continuously impairs patients' motor function, ultimately leading to the loss of self-care ability. For years, L-3,4-dihydroxyphenylalanine replacement therapy has been considered as the gold therapy of PD (Fahn et al., 2004). However, during the long-term treatment, L-dopa induced dyskinesia (LID) has gradually become another troublesome problem that aggravates the motor disability of Parkinson's patients (Warren Olanow et al., 2013; Marin et al., 2015). LID has been considered to be associated with the progressive dopamine loss in nigra striatum as well as the exogenous supplement of L-dopa (Bezard et al., 2001). Until now, the mechanisms underlying LID are still mysterious. Numerous studies identified the importance of the overactivation of the striatal dopamine D1 receptor (D1R) associated cascade (Corvol et al., 2004; Aubert et al., 2005; Guigoni et al., 2007) as the overreacted D1R expressed on the membrane of medium spiny neurons (MSNs) linking with Galphaolf proteins (Glatt and Snyder, 1993; Lebel et al., 2010) and adenylyl cyclase (AC; Santini et al., 2007), initially triggered exaggerated cyclic AMP formation and abnormal activation of cAMP-dependent protein kinase (PKA; Santini et al., 2010), resulting in the enhanced phosphorylation of downstream DA-and cAMP-regulated phosphorous-protein 32 kDa (DARPP-32) (Gerfen et al., 2002; Feyder et al., 2011). Phosphorylated DARPP-32 furtherly induced exaggerated phosphorylation of extracellular signal-regulated kinases 1/2 (ERK1/2), an important molecular event that was responsible for the initiation of nuclear gene expression and the final induction of LID (Corvol et al., 2004; Aubert et al., 2005). It is widely accepted that long-term intermittent dopamine stimulation could induce an aberrant activation of D1R and associated downstream cAMP/PKA/DARPP-32 signaling pathway in the direct pathway of striatum (Hemmings et al., 1984; Picconi et al., 2003). In contrast, continuous dopamine stimulation (CDS), featuring long-acting dopamine stimulation, has been evidenced in primates and Parkinson's patients to reduce dyskinesia effectively (Stocchi et al., 2005; Marin et al., 2006)

Recently, Fiorentini et al. (2011, 2013) reported a novel mechanism for D1R-mediated ERK1/2 activation in the rat striatum involving a tyrosine kinase, Shp-2. An interaction between D1R and Shp-2 was identified in their study and the D1R/Shp-2 interaction was evidenced to be essentially required for D1R-dependent phosphorylation of ERK1/2 (Fiorentini et al., 2011, 2013). Fiorentini et al. proposed that overreacted D1R induced the phosphorylation of Shp-2 (activate status) through cAMP/PKA/Src signaling pathway, finally resulting in the phosphorylation of ERK1/2. Striatal Shp-2 knockdown in 6-OHDA rat model remarkably attenuated the severity of LID (Fiorentini et al., 2016). They suggested that D1R/Shp-2 interface might become a potential target in the further clinical intervention of LID. However, the role of D1R/Shp-2 complex in LID development and its relationship with CDS were not well-clarified. Here we designed the study in which two different methods of L-dopa delivery (CDS vs. intermittent dopamine stimulation) were used to further identify: (1) whether the D1R/Shp-2/ERK1/2 signaling pathway was involved in the occurrence of LID; (2) whether CDS alleviated LID though preventing the over-expression of the D1R/Shp-2/ERK1/2 signaling pathway.

## Materials and methods

### Preparation of L-dopa methylester/bensarazide-loaded microspheres

The L-dopa/bensarazide microspheres (LBM) were prepared according to the oil-in-water emulsion solvent evaporation method. The procedure was the same as the method previously reported (Ren et al., 2011b; Rong et al., 2011; Yang et al., 2012; Zhao et al., 2013; Cai et al., 2014; Kang et al., 2014). L-dopa methyl ester and benserazide hydrochloride were purchased from Sigma-Aldrich (St. Louis, MO, USA).

### 6-hydroxydopamine (6-OHDA) models

Adult male rats (Sprague–Dawley, 180–220 g) were used in this study. Animal experiments were executed according to the guidelines of the National Institutes of Health (publication No. 80-23). All procedures were approved by the Institutional Review Board of Xinhua Hospital affiliated to Shanghai Jiao Tong University Medical School.

This model was made as described previously (Gan et al., 2015). Briefly, all rats were anesthetized by an intraperitoneal (i.p.) injection with ketamine (100 mg.kg^−1^). The rat was placed onto a stereotaxic frame (Narishige, Tokyo, Japan). 6-OHDA (Sigma Chemical Co., St. Louis, MO, USA) in a solution (in 0.9% saline with 0.02% ascorbic acid) was injected into the right medial forebrain bundle (MFB) of rats (6-OHDA concentration: 4 μg. μl^−1^. 6-OHDA total dose: 32 μg.rat^−1^). Two coordinates were as follows: at AP −3.7 mm, ML +1.7 mm, DV −7.8 mm; and at AP −4.4 mm, ML +1.2 mm, DV −7.8 mm. The tooth bar was set to −2.4 mm. Each site was injected with 16 ug 6-OHDA per rat. The sham animals were received the same surgery procedure except with an injection of a saline solution into the targeted sites.

### Drug treatment and behavioral assessment

Three weeks after injection, the 6-OHDA-lesioned rats were tested by the contralateral rotation assessment. The rats that exhibited rotational behaviors (at least seven turns per minute) following apomorphine injection (i.p.) (Sigma) at a dose of 0.25 mg.kg^−1^ were considered as successful rat models of PD (*n* = 38) and could be used for the following experiment.

The successful rat models of PD were randomly divided into four groups (Xie et al., 2014): (1) The L-dopa standard group (LS, *n* = 8): in this group, the rats were administrated twice-daily (9 a.m., 3 p.m.) with a L-dopa solution (L-dopa, 20 mg.kg^−1^, i.p. and bensarazide, 5 mg.kg^−1^, i.p.) for 21 days. (2) The L-dopa/bensarazide microspheres (LBM) low dose group (LBM-L, *n* = 8): in this group, the rats were subcutaneously administrated with LBM twice 1 day (9 a.m., 3 p.m.) per week for 3 weeks with L-dopa 20 mg.kg^−1^ plus beserazide 5 mg.kg^−1^. (3) The L-dopa/bensarazide microspheres (LBM) high dose group (LBM-H, *n* = 8): in this group, the rats were subcutaneously administrated with LBM twice 1 day (9 a.m., 3 p.m.) per week for 3 weeks with L-dopa 40 mg.kg^−1^ plus beserazide 10 mg.kg^−1^. (4) The PD group (*n* = 6): in this group, the 6-OHDA-lesioned rats were only administrated i.p. with 0.9% saline for 21 days. This group was used to assess function performance in Cylinder test. In addition, the sham group (*n* = 8) was administrated with 0.9% saline for 21 days.

### AIMs ratings

Abnormal involuntary movements (AIMs) would be assessed in all rats by an investigator who was blind to the experiment. The test would be performed once per day on day 2, day 7, day 12, day 17, and day 21. The detailed AIMs rating system was described in early reports (Xie et al., 2014). Briefly, the assessment of AIMs consists of three parts: the axial, the limb, and the orolingual movements (ALO AIMs; Lindenbach et al., 2011). For each part of the AIMs, the severity would be scored from 0 to 4 (Oh et al., 1999; Rylander et al., 2009). According to Cenci's method (Cenci et al., 1998), all rats were individually assessed for the ALO AIMs every 20 min during the 120 min of the pharmacological intervention. For each rat, the final score of each AIMs subtype was calculated by summing the 6 AIMs scores during 120 min. Therefore, the theoretical maximum score of each AIMs subtype would be 24 points. And the theoretical maximum score of the total ALO AIMs scores would be 72 points.

### Cylinder test

Here we used Cylinder test (Schallert et al., 2000) to assess functional improvement of the pharmacological intervention on the rat's impaired forelimb which was contralateral to the 6-OHDA-lesioned striatum. The test would be performed once per day on day 3, 8, 13, and 18. In the cylinder test, rats were individually placed into a transparent cylinder (20 cm diameter and 30 cm height) in a dimly lit room for 5 min. Such a movement would be recorded when the rat independently used its left or right forelimb to contact the wall during a full rear to initiate a weight-shifting movement or to regain center of gravity while moving laterally in a vertical posture. Functional improvement of the rat's impaired forelimb was assessed by the percentage use of the impaired forelimb relative to the total number of limb use movements.

### Striatal protein extraction

Rats were sacrificed in day 22 (24 h after last injection) by decapitation to prepare the total protein extraction of lesioned-striatum. The method of extraction was described previously (Gan et al., 2015). Five striatal tissues in every group (LS, LBM-L, LBM-H, sham group) were used to pellet the cytosol fractions. Striatal tissue was dissected and frozen in liquid nitrogen, then homogenized by sonication in RIPA buffer (50 Mm Tris, 150 mM NaCl, 0.1% sodium dodecyl sulfate, 1% Nonidet P-40, 1 mM ethylenediaminetetraacetic acid, a protease inhibitor cocktail and 2 mM phenylmethylsulfonyl fluoride). The homogenate was centrifuged at 4°C for 10 min at 700 g and the supernatant was collected. The pellet which contained large debris or nuclei was discarded. To pellet membrane-enriched proteins, the rest three striatal tissues in every group were homogenized and centrifuged using Membrane Protein Extraction Kit (Thermo Scientific, Waltham, MA, USA) according to the manufacturer's instructions to pellet the membrane fraction (Song et al., 2016). Protein concentrations were determined in the supernatant using a bicinchoninic acid (BCA) assay kit (Pierce, Rockford, IL, USA).

### Western blot analysis

Samples containing equivalent amounts of protein (30 μg) were electrophoresed on 10% sodium dodecyl sulfate-polyacrylamide gel. Proteins were electro-transferred to polyvinylidene fluoride (PVDF) membrane for immunoblotting in Tris-glycine transfer buffer. Then, membranes were blocked in blocking buffer with 5% non-fat dry milk in TBS-Tween-20 for an hour at room temperature. Membranes were incubated at 4°C overnight with antibody recognizing Shp-2 (1:2,000) (Proteintech Group, Inc., Resemont, IL, US); phospho-Shp-2 (1:1,000) (Signalway Antibody LLC, College Park, MD, USA); Src (1:2,000) (Santa cruz technology, CA, USA); phospho-Src (1:1,000) (Cell signaling, MA, USA); phospho-DARPP-32 (Thr34) (1:1,000) (Absin Bioscience Inc., Shanghai, CHN); DARPP-32 (1:1,000) (Cell Signaling Technology, Boston, MA, USA); ERK1/2 (1:2,000) (Cell signaling, MA, USA); phospho-E RK1/2 (1:1,000) (Cell signaling, MA, USA); D1R (1:1,000) (Millipore, MA, USA); GAPDH (1:5,000) (Proteintech Group, Inc, Resemont, IL, US). Subsequently, the membranes were washed extensively with TBS-T and incubated with horseradish peroxidase-conjugated (HRP-conjugated) anti-rabbit or anti-mouse IgG (1:2,000) (Cell signaling, MA, USA) for an hour at room temperature. Visualization of immunoreactive proteins was achieved by using the enhanced chemiluminescence detection system (Millipore). Bands of interest were analyzed quantitatively using the Quantity One Software (Bio-Rad).

### Statistical analysis

Data were expressed as the mean ± standard deviation (*SD*). Non-parametric methods were used in the analysis of the behavioral data. Kruskal–Wallis test was used for multiple comparisons of behavioral data of each observation among different groups. The *post-hoc* test was evaluated with the Nemenyi test. Wilcoxon test was used for comparison of AIMs rating in each of the groups between day 2 and day 21 of the treatment. One-way analysis of variance was used for comparison of biochemical data among groups (*n* ≥ 3). Statistical significance was set at *p* < 0.05.

## Results

### Effect of administration of LBM on AIMs in 6-OHDA-lesioned rats

**Figure 1:**
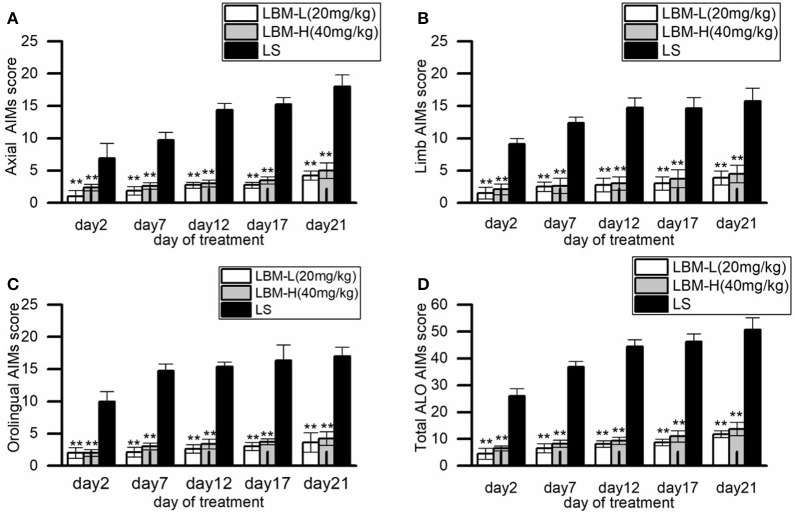
Behavioral characteristics of the 6-OHDA-lesioned rats during 21 days of treatment with LBM-L (20 mg/kg), LBM-H (40 mg/kg), and L-dopa plus benserazide standard (LS). **(A–D)** Measures of Axial **(A)**, Limb **(B)**, Orolingual **(C)**, and the total ALO AIMs **(D)** in the rats treated with LBM-L, LBM-H, or LS group on each assessment during 21 days of treatment. During the period, intermittent administration of L-dopa induced a robust increase of the total ALO AIMs assessment and each of the sub items in 6-OHDA-lesioned rat models of PD, with apparent dyskinetic movements. In contrast, this phenomenon was not observed in 6-OHDA-lesioned rat models of PD treated with LBM (the LBM-L and LBM-H groups). The scores of total ALO AIMs assessment and its sub items in 6-OHDA-lesioned rat models of PD of LS group were all significantly higher than 6-OHDA lesioned rat models of PD treated with LBM (the LBM-L and LBM-H groups) in all assessments (*p* < 0.01). Moreover, the scores of total ALO AIMs assessment and its sub items in LS group were significantly different between day 2 and day 21 (*p* < 0.01). No difference was found in all items of AIMs assessments between the rats in the LBM-L group and rats in the LBM-H group. Data are presented as mean ± *SD*; ^**^p < 0.01 vs. LS group (*n* = 8 per group).

During 21 days of treatment with L-dopa plus benserazide (LS group), the 6-OHDA-lesioned rats gradually presented with apparent dyskinetic movements. A continuous increase trend was found in the scores of ALO AIMs assessments during the period of administration. The mean axial AIMs score significantly increased from (6.87 ± 2.36) on day 2 to (18.00 ± 1.85) on day 21 (*Z* = −2.226, *p* = 0.026; Figure 1A), and the mean limb AIMs score significantly increased from (9.12 ± 0.84) on day 2 to (15.75 ± 2.04) on day 21 (*Z* = −2.214, *p* = 0.027; Figure 1B). The mean orolingual AIMs score also significantly increased from (10.00 ± 1.51) on day 2 to (17.00 ± 1.41) on day 21 (*Z* = −2.214, *p* = 0.027; Figure 1C). Hence, the total ALO AIMs score increased from (26.00 ± 2.71) on day 2 to (50.75 ± 4.44; *Z* = −2.207, *p* = 0.027) on day 21 (shown in Figure 1D).

Compared with the rats of the LS group, the 6-OHDA-lesioned rats showed a slight increase of AIMs scores during the treatment of LBM (both the LBM-L and LBM-H dose). The total ALO AIMs score in the LBM-L group increased from (4.50 ± 1.98) on day 2 to (11.75 ± 1.30) on day21 (*Z* = −2.214, *p* = 0.027 vs. day 2, Figure 1D) and the sum of ALO AIMs score in the LBM-H group increased from (6.50 ± 0.89) on day 2 to (12.75 ± 2.49) on day 21 (*Z* = −2.201, *p* = 0.028 vs. day 2, Figure 1D). The AIMs scores of all assessments in the LBM-L and LBM-H group were significantly less than in the LS group (*p* < 0.01, Figures 1A–D). The rats treated with LBMs did not present with obvious dyskinesia in symptoms. Furthermore, no difference was found in all items of AIMs assessments in rats between the LBM-L group and LBM-H group (*p* > 0.05, Figures 1A–D).

### Effect of administration of LBM on motor function in 6-OHDA-lesioned rats

**Figure 2:**
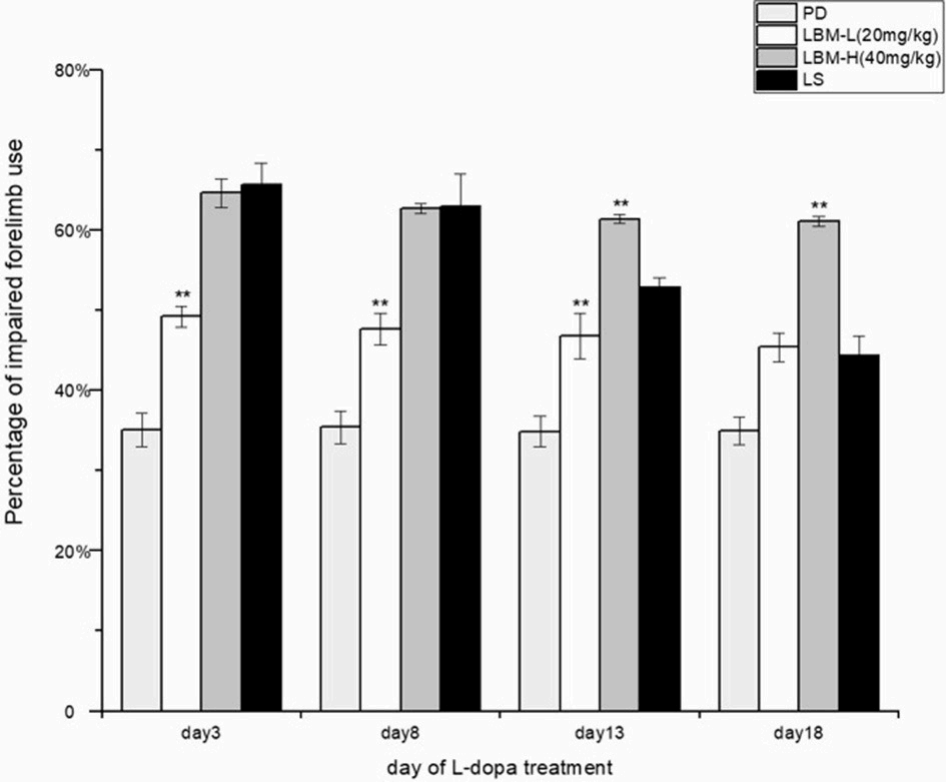
Motor improvement of 6-OHDA lesioned rat models of PD treated with LBM-L (20 mg/kg) (*n* = 8), LBM-H (40 mg/kg) (*n* = 8), LS (*n* = 8), or saline (PD group) (*n* = 6) was assessed through cylinder tests on day 3, 8, 13, and 18 of the treatment. The unilaterally 6-OHDA-lesioned rats preferred to touch the inner wall of the cylinder with the impaired forelimbs more frequently during the treatment with intermittent L-dopa administration or LBM, compared with the 6-OHDA rats treated with 0.9% saline (PD group) (*p* < 0.01). On day 3 and day 8 of the treatment, no difference was found in the rats' preference in touching the inner wall with the impaired forelimbs in cylinder tests between the LS and LBM-H group. However, as the treatment continued, motor improvement of the 6-OHDA lesioned rats' impaired forelimbs in the LS group was gradually weakened by the occurrence of dyskinetic movements. In contrast, motor improvement kept stable in both the LBM-L and LBM-H group during 21 days of treatment. Although, 6-OHDA-lesioned rats of the LS group used the impaired forelimbs more frequently than rats of the LBM-L group in cylinder tests on day 3, day 8, and day 13 of treatment (*p* < 0.05), the difference was not significant between the two groups on day 18 of treatment. Remarkably, rats of the LBM-H group used its impaired forelimbs more frequently in the cylinder test than rats of the LS group after day 13 of treatment (including day 13). In addition, rats of the LBM-H group demonstrated a significantly better functional improvement than rats of the LBM-L group in all cylinder tests during 21 days of treatment (*p* < 0.05). Data are presented as mean ± *SD*; ^**^p < 0.01 vs. LS group.

During 21 days of treatment, functional improvement of the impaired forelimbs was observed in the rats of different groups. During the treatment, the 6-OHDA rats of the LS group and LBM group (LBM-L and LBM-H) preferred to use its impaired forelimbs (contralateral to the lesion) more frequently than the rats of PD group (treated with 0.9% saline; *p* < 0.01). However, functional improvement of the LS group presented with a decline tendency. In contrast, it kept stable in the 6-OHDA rats treated with LBM for 21 days, both in the LBM-L group and in LBM-H group. The 6-OHDA rats of the LS group used the impaired forelimbs touching the inner wall in the cylinder tests more frequently than those of the LBM-L group on day 3, 8, and 13 of treatment (*p* < 0.01). However, the difference was not significant between the two groups on day 18 of treatment (*p* > 0.05). Interestingly, rats of the LBM-H group demonstrated more apparent preference with the impaired forelimbs in the cylinder test than rats of the LS group after day 13 of treatment (including day 13; *p* < 0.01). In addition, rats of the LBM-H group also demonstrated more apparent preference with the impaired forelimbs in the cylinder test than rats of the LBM-L group during 21 days of treatment (*p* < 0.05; Figure 2).

### Changes in expression of membrane-D1R after administration of LBM in 6-OHDA-lesioned striatum

**Figure 3:**
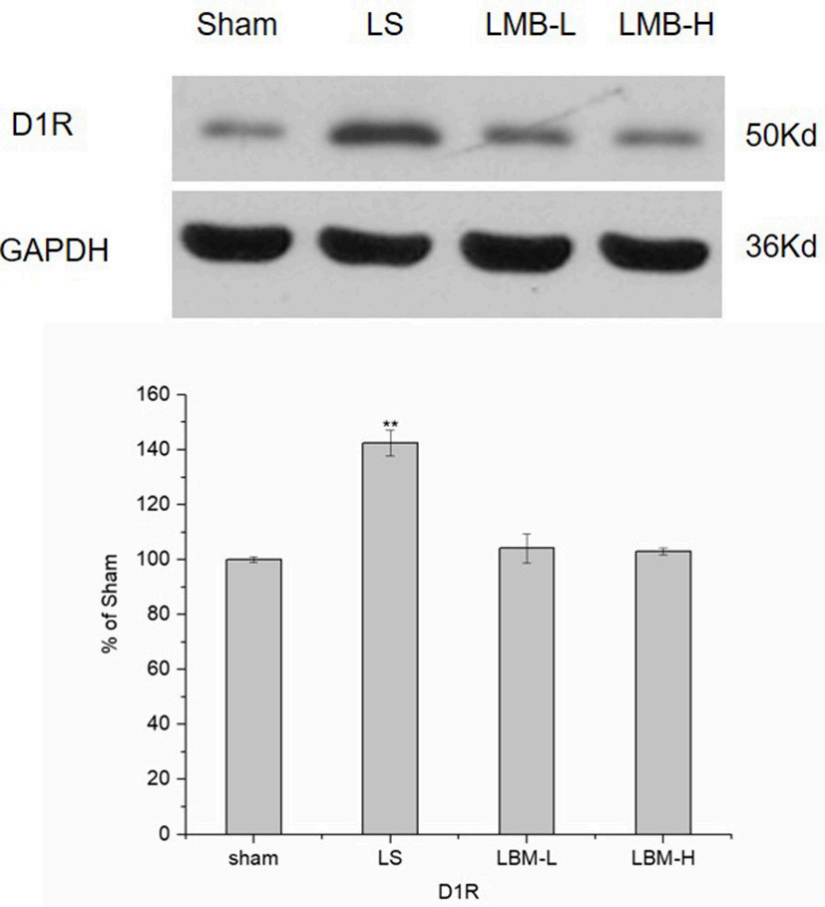
Changes in expression of membrane-D1R extracted from the rat's ipsilateral striatum after 21 days of administration. The expression of membrane-D1R in the LS group was significantly higher than that in the LBM group (LBM-L and LBM-H) and sham group (*p* < 0.01). There was no difference among the LBM-L, LBM-H, and the sham group. The membrane-D1R levels expressed relative to GAPDH levels. The data are expressed in terms of means ± *SD* and expressed as a percentage of the sham group. ^**^p < 0.01 vs. sham group (*n* = 3 per group).

As shown in the Figure 3, intermittent L-dopa administration induced a significant increase of the striatal membrane D1R protein level in the 6-OHDA-lesioned striatum of rats in the LS group. The expression of membrane-D1R of rats in the LS group was significantly higher than that in the LBM group (LBM-L and LBM-H) and sham group (*p* < 0.01). In contrast, LBM treatment didn't change the striatal membrane D1R protein level. No difference was found in the expression of membrane-D1R among the LBM-L, LBM-H, and sham group (Figure 3).

### Changes in expression of Shp-2, Src after administration of LBM in 6-OHDA-lesioned striatum

**Figure 4:**
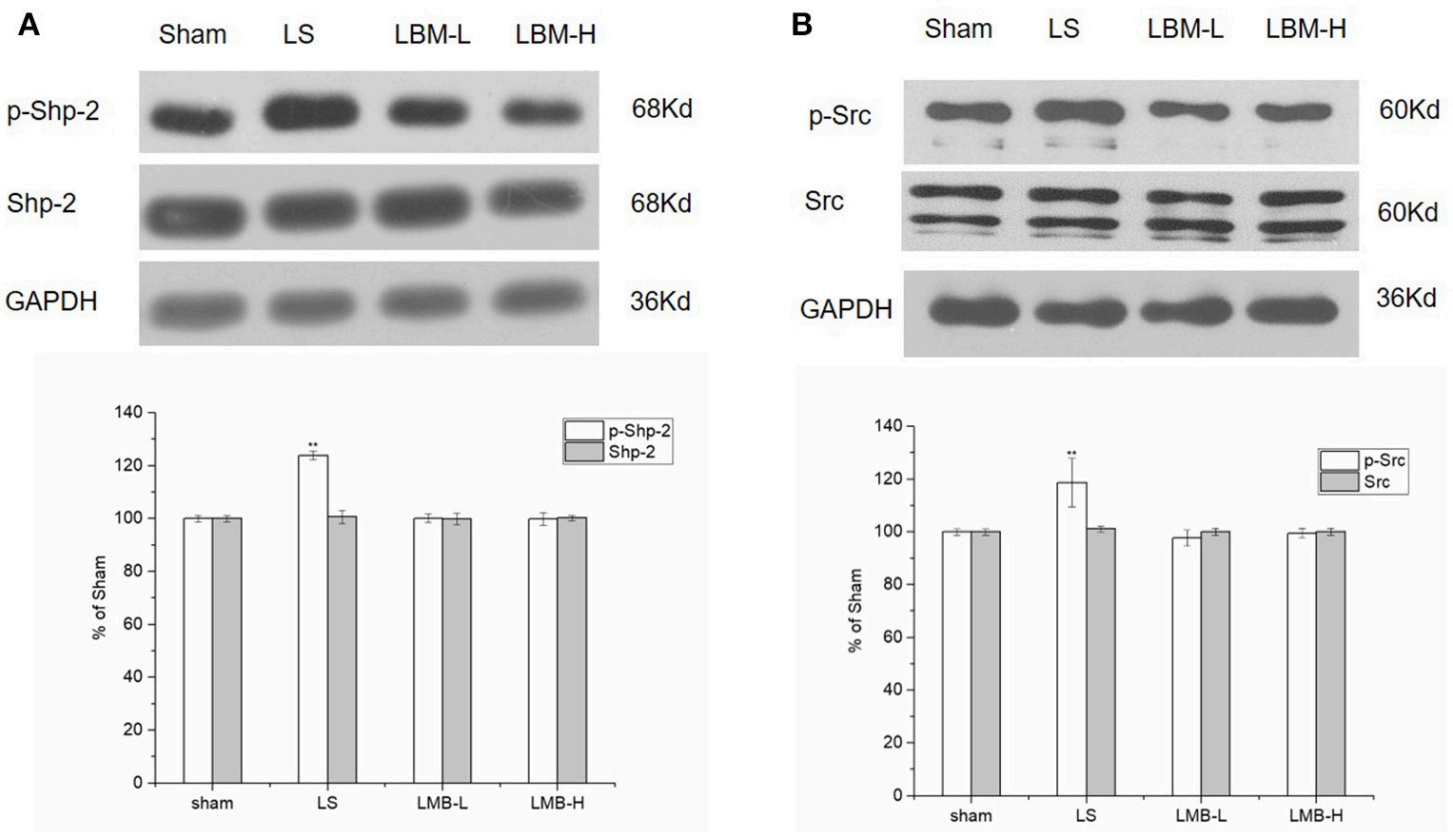
Changes in expression of phospho-Shp-2, Shp-2 **(A)**, and phospho -Src, Src **(B)** after either intermittent or continuous L-dopa administration in 6-OHDA-lesioned striatum for 21 days. **(A)** The phosphorylated Shp-2 level at Tyr542 increased significantly in the LS group compared with the sham, LBM-L, and LBM-H group (*p* < 0.01). No significant difference was found in the phosphorylation level of Shp-2 among the LBM-L and LBM-H and sham group (*p* > 0.05). The total amount of Shp-2 remained stable in all groups (*p* > 0.05). **(B)** Phosphorylated Src level at Ser17 increased significantly in LS group compared with the sham, LBM-L and LBM-H group (*p* < 0.01). No significant difference was found in the phosphorylation level of Src among the LBM-L and LBM-H and sham group (*p* > 0.05). The total amount of Src remained unchanged in all groups (*p* > 0.05). All above protein levels expressed relative to GAPDH levels. The data are expressed in terms of means ± *SD* and expressed as a percentage of the sham group. ^**^p < 0.01 vs. sham group (*n* = 5 per group).

Shp-2 activation was reported to be crucial for the D1R mediated phosphorylation of ERK1/2 (Fiorentini et al., 2011, 2013, 2016). In this part of our study, we wanted to know the effect of CDS on Shp-2 Tyr 542 phosphorylation. We found that intermittent L-dopa administration resulted in a robust increase in Tyr 542 phosphorylation level of Shp-2 in the LS group. Quantitative analysis indicated that the level of Shp-2 phosphorylation was significantly elevated in the LS group compared to the LBM group (LBM-L and LBM-H) and sham group (*p* < 0.01). However, Shp-2 phosphorylation remained unchanged after the LBM treatment (LBM-L and LBM-H group; Figure 4A). No significant difference was found in the phosphorylation level of Shp-2 among the LBM-L, LBM-H, and sham groups. The total amount of Shp-2 remained stable in all groups (Figure 4A). Our resulted showed that intermittent L-dopa administration induced an enhanced Shp-2 phosphorylation; however, the LBM treatment prevented this hyperphosphorylation of Shp-2.

Tyrosine kinase Src has been evidenced to play an important role in the hormonal regulation of ERK via cyclic AMP (Ravetch and Lanier, 2000; Ren et al., 2004). Here we also evaluated the activation of Src in rats treated with different methods. The phosphorylation of Src at residue serine 17 (S17) was considered as activation of Src (Collett et al., 1979). We found that a robust increase of phosphorylated Src in the lesioned striatum of rats after 21 days of intermittent L-dopa administration. The phosphorylation level of Src was significantly elevated in the LS group compared to the LBM group (LBM-L and LBM-H) and sham group (*p* < 0.01; Figure 4B). In contrast, the LBM treatment did not lead to an increasing Src activity in the lesioned striatum of rats as no significant difference of phosphorylated Src was found between the LBM group and sham group (Figure 4B). Meanwhile, we also evaluated the effect of different dose of LBM on the targeted proteins. There was no significant difference on the expression of Src phosphorylation between the LBM-L group and LBM-H group (Figure 4B). The level of Src still remained unchanged in all groups (Figure 4B).

### Changes in expression of DARPP-32 after administration of LBM in 6-OHDA-lesioned striatum

**Figure 5:**
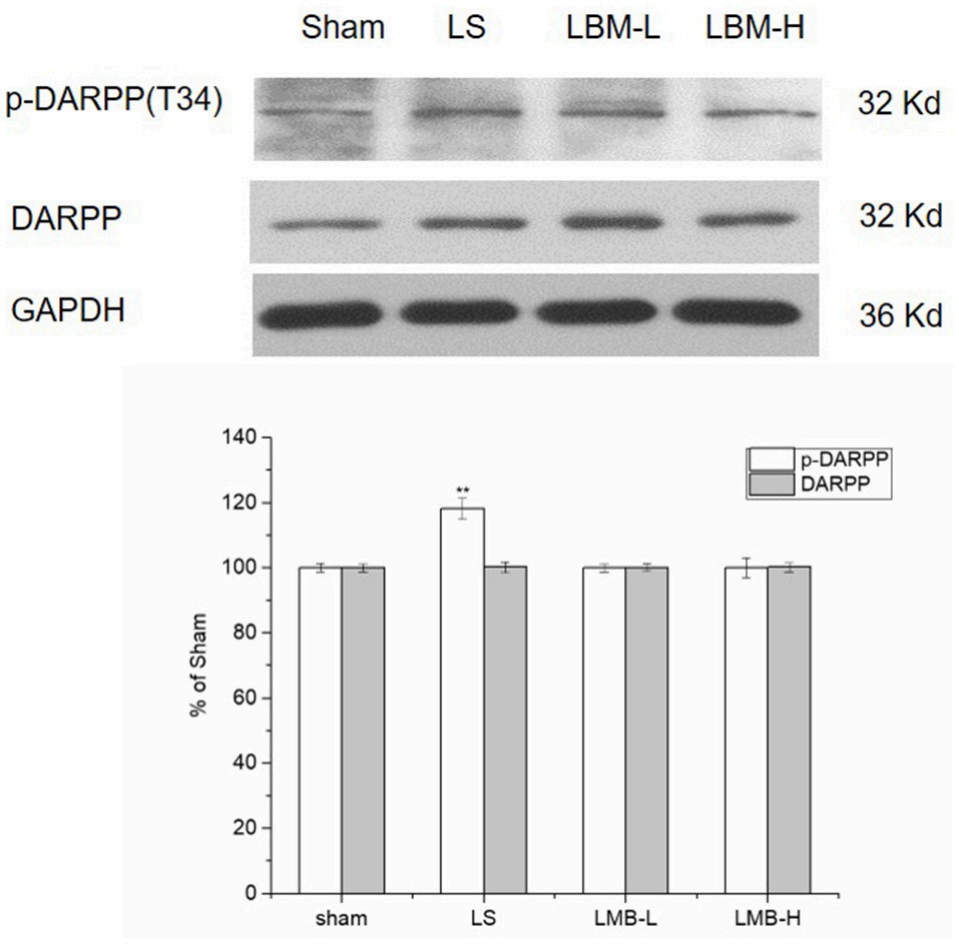
Changes in expression of phospho-DARPP-32 (Thr34), DARPP-32 after either intermittent or continuous L-dopa administration in 6-OHDA-lesioned striatum for 21 days. The phospho-DARPP-32 (Thr34) level in the LS group was much higher than the sham, LBM-L group and LBM-H group (*p* < 0.01). No difference was found in the phospho-DARPP-32 (Thr34) level among the LBM-L and LBM-H and sham group (*p* > 0.05). The total amount of DARPP-32 remained stable in all groups (*p* > 0.05). All above protein levels expressed relative to GAPDH levels. The da0074a are expressed in terms of means ± *SD* and expressed as a percentage of the sham group. ^**^p < 0.01 vs. sham group (*n* = 5 per group).

DARPP-32 phosphorylation has been considered as an important biomarker for LID. Since the previous studies identified an enhanced phosphorylation of DARPP-32 at residue threonine 34 (Thr34) in the PD rats developing LID, we tested the phosphorylation of DARPP-32 at Thr34 in the rats treated with different methods. We detected a significant increase of the phosphorylated DARPP-32 in the lesioned striatum of rats after 21 days of intermittent L-dopa administration. The phosphorylation level of DARPP-32 was significantly higher in the LS group compared to the LBM group (LBM-L and LBM-H) and sham group (*p* < 0.01; Figure 5). However, no difference was found in the level of phosphorylated DARPP-32 between the LBM group and sham group (Figure 5). Moreover, the phosphorylation level of DARPP-32 remained the same between the LBM-L group and LBM-H group (Figure 5). The level of DARPP-32 remained unchanged in all groups (Figure 5).

### Changes in expression of ERK1/2 after administration of LBM in 6-OHDA-lesioned striatum

**Figure 6:**
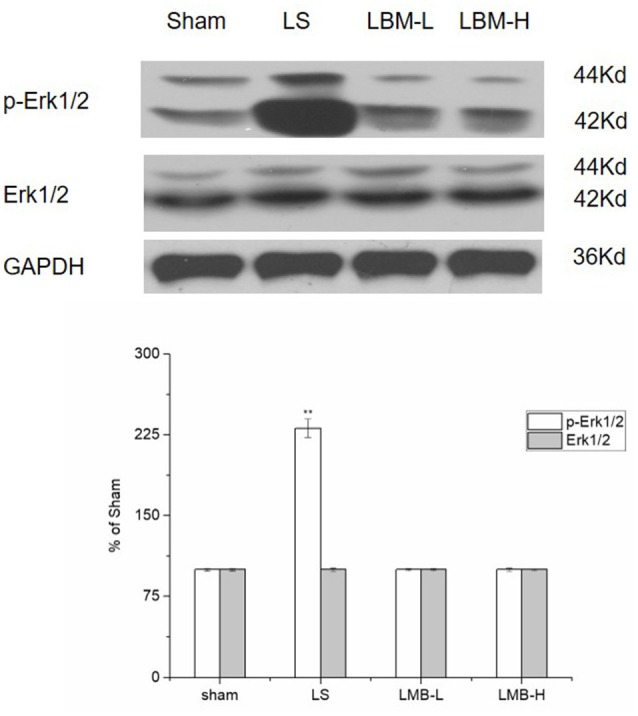
Changes in expression of phospho-ERK1/2, ERK1/2 after either intermittent or continuous L-dopa administration in 6-OHDA-lesioned striatum for 21 days. The expression of phospho-ERK1/2 in the LS group significantly increased compared with the sham, LBM-L and LBM-H group (*p* < 0.01). In contrast, no difference was found in the expression of phospho-ERK1/2 among the sham, LBM-L and LBM-H group (*p* > 0.05). The level of ERK1/2 protein still remained unchanged in all groups (*p* > 0.05). All above protein levels expressed relative to GAPDH levels. The data are expressed in terms of means ± *SD* and expressed as a percentage of the sham group. ^**^p < 0.01 vs. sham group (*n* = 5 per group).

The phosphorylation of ERK1/2 plays a critical role for the initiation of nuclear gene expression and the final induction of LID. We compared the level of EKR1/2 and its phosphorylation among different treatment groups. Our data found that intermittent L-dopa administration activated ERK1/2 protein by significantly increasing the phosphorylation of ERK1/2 in the lesioned striatum of the LS group. The level of ERK1/2 phosphorylation was significantly elevated in the LS group compared to the LBM group (LBM-L and LBM-H) and sham group (*p* < 0.01; Figure 6). In contrast, the overexpression of ERK1/2 phosphorylation was not observed in the rats treated with LBMs. No difference was found in the level of ERK1/2 phosphorylation among the LBM-L, LBM-H, and sham group. The level of ERK1/2 protein still remained unchanged in all groups (Figure 6).

## Discussion

Our study confirmed that chronic intermittent stimulation of L-dopa resulted in a persistent overreaction of D1R/Shp-2/ERK1/2 signaling pathway, while stable and moderate release of L-dopa by LBM significantly prevented such an overreaction and effectively reduced the total AIMs score in the 6-OHDA-lesioned rat. This indicated that overactivation of D1R/Shp-2/ERK1/2 signaling pathway was required for the induction of LID in the 6-OHDA-lesioned rat models of PD.

First, our study found that LBM treatment had more advantages than intermittent L-dopa administration in aspect of inducing less dyskinesia and alleviating parkinsonian symptoms in 6-OHDA-lesioned rats. The LBM were characterized to imitate CDS by a sustained and stable release of the loaded drug (Ren et al., 2011a,b). The L-dopa and benserazide loaded in the poly (lactic-co-glycolic acid) have been evidenced to be released to the plasma in a gradual manner. 16.7% of the loaded drugs released at day 1 and about 99% of loadings released up to 17 days (Ren et al., 2011b). The findings in this study were consistent with the previous studies of our team (Ren et al., 2011b; Xie et al., 2014). The advantage of LBM was mainly attributed to its special pharmacokinetic characteristics. The stable release effectively prevented a pulsatile stimulation to dopaminergic receptors in the striatum, resulting in the less occurrence of dyskinesia. Moreover, our results indicated that LBM treatment improved the rats' parkinsonian symptoms in a dose-dependent manner, as the dose of L-dopa in the LBM-H group (40 mg.kg^−1^) was doubled, the LBM-H group (40 mg.kg^−1^) played a more apparent anti-parkinsonian role than the LBM-L group (20 mg.kg^−1^), without a significant increase of AIMs scores during the observations. In contrast, the anti-parkinsonian effect of intermittent L-dopa administration presented with a decline tendency during the period of treatment, indicating that the treatment benefit of intermittent administration was gradually counteracted by the occurrence of dyskinesia. Therefore, our results supported that LBM treatment, imitating CDS, had an apparent superiority on parkinsonian interventions in rodent experiments.

Then we focused on the molecular mechanism of CDS on striatal DA receptor in LID to clarify the association of CDS and LID. To our knowledge, the abnormal overexpression of striatal DA receptors (especially D1 receptor) is closely associated with the induction of LID (Glatt and Snyder, 1993; Guigoni et al., 2007; Fiorentini et al., 2011, 2013). Chronic L-dopa stimulation in a pulsatile manner induced the aberrant overresponse of D1R in the MSNs of the dopamine-depleted striatum, featuring a robust increase of D1R located on the membrane of MSNs (Berthet et al., 2009). According to the previous studies (Hemmings et al., 1984; Picconi et al., 2003), the overexpressed D1R, furtherly through activating the cAMP/PKA/DARPP-32 signaling pathway, resulted in the phosphorylation of ERK1/2 which was a crucial part of LID. Currently, a novel signal pathway involving D1R/Shp-2 interaction was proposed in the development of LID (Fiorentini et al., 2011, 2013, 2016). Activation of Shp-2 in striatal D1R/Shp-2 complex was required for D1R-dependent signaling transmission to the phosphorylation of ERK1/2. D1R interaction with Shp-2 leaded to Shp-2 tyrosine phosphorylation. Aberrant activation of the D1R-Shp-2complex leaded to an enhanced ERK1/2 phosphorylation which was an important molecular event underlying LID in the 6-OHDA-lesioned rat model of PD (Fiorentini et al., 2013, 2016). According to the above viewpoint, the corresponding molecular changes would be observed in the striatum neurons of PD rodent models with LID.

Our results showed that intermittent L-dopa stimulation induced a robust increase of membrane bound D1R as well as an enhanced phosphorylation of Shp-2 and ERK1/2 in the 6-OHDA-lesioned striatum. Accordingly, these rats developed apparent dyskinesia with the obvious increase of the total AIMs scores. In contrast, the similar change was not detected in rats treated with LBM. The expression of membrane-D1R and phosphorylation of Shp-2 in the LBM group (both high-dose and low-dose) was significantly lower than that in the LS group developing dyskinesia. Equally, the expression of phospho-ERK1/2 was reduced in LBM group, compared with that in LS group. These data indicated that there was a significant difference in the activation of D1R/Shp-2/ERK1/2 signaling pathway underlying different types of L-Dopa administration. D1R/Shp-2 complex was preserved and kept a persistent overexpression after the chronic intermittent L-dopa stimulation, compared with CDS.

Shp-2 activation is the key to the activation of D1 receptors. Shp-2 is a widely expressed protein tyrosine phosphatase, featuring a typical SH2 domains contained at its C terminal (Neel et al., 2003; Poole and Jones, 2005). Shp-2 would be activated though the tyrosine phosphorylation contained at the C terminal SH2 domain. The phosphorylation would be activated when the SH2 domain binds a proper partner protein which usually contains one or more “immuno-receptor tyrosine-based inhibitory motif” (ITIMs; Ravetch and Lanier, 2000). Currently, Fiorentini et al. confirmed the ITIM domain in the sequence of the D1R (VTY214TRI) of HEK 293 cells, the striatum neurons of normal and parkinsonian rats with dyskinesia (Fiorentini et al., 2011). Furthermore, Shp-2 was evidenced to be interacted with D1R through the ITIM domain, and the interaction would be required for D1R mediated phosphorylation of ERK1/2. In dopamine-depleted striatum of rats with dyskinesia, a robust phosphorylation of ERK1/2 and Shp-2 was both confirmed in the lesioned sides (Fiorentini et al., 2013). This hyperphosphorylation could be enhanced by the D1 agonist (SKF38393) or prevented by the D1 antagonist (Sch23390; Fiorentini et al., 2013). In addition, the phosphorylation of ERK 1/2 activated by SKF 81297 (D1 agonist) could not be induced in Shp-2 silenced striatal neurons (Fiorentini et al., 2011). All these studies supported that D1R/Shp-2 complex and the activation of Shp-2 were crucial for the phosphorylation of ERK1/2 and the occurrence of LID.

In addition to hyperphosphorylation of Shp-2, a significant hyperphosphorylation of Src was confirmed in the 6-OHDA-lesioned striatum of the dyskinesia rats treated with intermittent stimulation of L-dopa in our study. In contrast, there was no evident phosphorylation of Src in the PD rats treated with LBM. The study showed that tyrosine kinase Src was required in the activation of Shp-2 as the selective Src inhibitor (PP2) prevented the phosphorylation of Shp-2 induced by SKF81297 (Fiorentini et al., 2011). The association between Shp-2 and Src was not fully clarified, however, previous studies showed that Shp-2 activation was required for the dephosphorylation of the inhibitory tyrosine on Src family kinases (SFK), probably by controlling Csk tyrosine kinase recruitment and thus for SFK activation (Ren et al., 2004). Then activated SKF furtherly participated in the activation of the Ras/ ERK cascade (Zhang et al., 2004). Therefore, Shp-2 and Src might be required for the D1 mediated activation of ERK1/2 through a reciprocal regulation. Our data showed that intermittent dopamine stimulation resulted in a simultaneous hyperactivation of Shp-2 and Src in 6-OHDA-lesioned striatum, while CDS could prevent the overreaction of Shp-2 and Src. This suggested that Shp-2-Src synergistical regulation would be crucially involved in D1R-mediated activation of ERK1/2.

We have mentioned that, in the classical D1R mediated signaling transmission, activated PKA induced an exaggerated phospho-DARPP-32, resulting in the phosphorylation of ERK1/2. DARPP-32, expressed in the MSNs of the striatum, is an important downstream effector of D1R/cAMP/PKA signaling pathway (Gerfen et al., 2002; Feyder et al., 2011). Phosphorylated DARPP-32 at Thr34 induced by PKA converts DARPP-32 into an inhibitor of protein phosphatase-1 (PP-1; Hemmings et al., 1984). By inhibiting PP-1, DARPP-32 furtherly modifies the state of phosphorylation of protein kinases and protein phosphatases that control the state of phosphorylation of ERK (Valjent et al., 2005). Our study also observed an enhanced DARPP-32 phosphorylation at Thr34 in the lesioned striatum of rats with LID induced by intermittent L-dopa stimulation. This was not detected in the rats treated with CDS. This phenomenon indicated that a difference in the DARPP-32 phosphorylation level between two different types of L-dopa administration. Hyperphosphorylation of DARPP-32 was required for dyskinesia induced by intermittent L-dopa stimulation. Therefore, the co-activation of D1R/Shp-2 complex and D1R/PKA/DAPRR-32 signaling pathway might be involved in the ERK1/2 phosphorylation and the occurrence of LID. Interestingly, PKA was evidenced to be also required for D1R mediated activation of Shp-2 as the PKA inhibitor (H89) blocked phosphorylation of Shp-2 in the striatal neurons induced by the D1 agonist (SKF81297) (Fiorentini et al., 2011). An opinion was proposed as activated D1R first induced activation of PKA and activated PKA furtherly induced a significant phosphorylation of Shp-2 and DARPP-32 (Fiorentini et al., 2011). The activated Shp-2 and DARPP-32 then synergistically acted to trigger and maintain the activation of ERK1/2, finally resulting in the occurrence of LID. However, this opinion might require more convincing evidence to support and might be testified in our further studies.

## Conclusion

Taken together, our data presented that using L-dopa/benserazide microspheres (LBM) treatment, imitating CDS, might effectively alleviate LID through preventing the aberrant activation of D1R associated signaling cascade. CDS played its role through preventing the overreaction of D1R/Shp-2/ERK1/2 signaling pathway. Our results further confirmed that the activation of D1R/Shp-2/ERK1/2 was required in the mechanism of LID. Moreover, LBM treatment was capable to avoid the overresponse of D1R/Shp-2 complex, preventing the phosphorylation of ERK1/2 and the occurrence of LID. These results might provide a novel direction for the mechanisms of LID development and a new intervention target for clinical investigations.

## Ethics statement

All animals survived under Specific Pathogen Free (SPF) environment in a laboratory within the animal facility located at the Xinhua Hospital affiliated to Shanghai JiaoTong University, School of Medicine. All mice were housed under standardized laboratory conditions and monitored to observe changes in ordinary conditions and activities. Animal care and use were in accordance with the guidelines established by the Administration of Affair Concerning Laboratory Animals for Shanghai JiaoTong University, the National Institutes of Health Guide for care and Use of Laboratory Animals (GB14925-2010) and the Regulations for the Administration of Affairs Concerning Experimental Animals (China, 2014).

## Author contributions

YW, NW, LS, XW, ZL, WY, and JG participated in its design, searched databases, extracted and assessed studies and helped to draft the manuscript. WY and JG conceived the initial idea and the conceptualization, participated in the data extraction and analysis, and revised the manuscript. All authors read and approved the final manuscript.

### Conflict of interest statement

The authors declare that the research was conducted in the absence of any commercial or financial relationships that could be construed as a potential conflict of interest.
